# Multi-marker approach for the evaluation of environmental impacts of APACS 50WG on aquatic ecosystems

**DOI:** 10.1007/s10158-020-00254-2

**Published:** 2020-11-16

**Authors:** Dávid Somogyvári, Ágnes Vehovszky, Anna Farkas, Réka Horváth, János Győri

**Affiliations:** 1 Department of Experimental Zoology, Balaton Limnological Institute, MTA Centre for Ecological Research, Tihany, 8237 Hungary; 2 Department of Engineering, University of Pannonia, Veszprém, 8200 Hungary

**Keywords:** *Dikerogammarus villosus*, Neonicotinoid, APACS 50WG, Insecticide, Behavioural responses, Swimming activity, MXR

## Abstract

Neonicotinoids are the most widely used synthetic insecticides in the world. These insecticides are widely distributed in the ecosystem, indicating that more attention should be paid to the potential risks regarding their use in agriculture. Due their intensive use, non-target species in the environment are also exposed to their putative effects. Within acute exposure trials, the time related effect of sublethal dose of the neonicotinoid preparation APACS 50 WG was investigated on swimming behaviour and the multi-xenobiotic resistance system (MXR) activity, as a first line defence pathway of adult *Dikerogammarus villosus*. Results showed that treated animals manifested an increased swimming activity. Exposed animals were monitored by the rhodamine B accumulation assay, and APACS 50 WG exerted distinct changes in the MXR activity as well. Our results suggested that application of neonicotinoid at a low concentration (3.9 ng/l) contributed to the activation of locomotor activity and at the same concentration range the transmembrane transport mechanisms was altered too.

## Introduction

Neonicotinoids are considered among the most important classes of pesticide active ingredients currently used in agricultural crop protection. Neonicotinoids mimic the action of acetylcholine (ACh), one of the main excitatory neurotransmitters of the central nervous system and the primary mechanism of action is their strong binding to the postsynaptic nicotinic acetylcholine receptors (nAChRs) (Tomizawa and Casida 2005) showing a more selective pharmacological/toxicological profile to arthropod than vertebrate receptors.

The majority of scientific publications focus on imidacloprid, which was the first and widely applied neonicotinoid, but the most commonly used, thiamethoxam, and its metabolite clothianidin have received less attention. In the American cockroach, *Periplaneta americana* (L.), topical application of the clothianidin induced behavioural responses (Tan et al.), and clothianidin was highly effective eliciting symptoms of poisoning. Cockroach locomotor activity also was changed during clothianidin treatment (Benzidane et al. 2010).

Recent detection of clothianidin in surface waters has raised interest in characterizing the potential impacts of this insecticide to aquatic organisms as well. Evaluation of locomotor activity as a potential biomarker of exposure of environmental stressors was suggested to monitor freshwater ecosystems (Wallace and Estephan 2004; Felten et al. 2008). It has already been proven that the multixenobiotic resistance system (MXR) is activated by the effect of various neonicotinoids in aquatic invertebrates (Vehovszky et al. 2018).

The aim of our study was to evaluate the effect of a formulated neonicotinoid *APACS* 50 WG using *Dikerogammarus villosus* as a model animal. We conducted locomotor activity test to detect behavioural changes following APACS 50WG exposure, and MXR analysis were performed focusing on the expression of the first line defence pathway.

## Methods

### Collection and maintenance of animals

Adult (5–8 mm length) killer shrimps, *D. villosus,* which is an invasive species populating Lake Balaton, were collected locally (Tihany, Hungary) from the littoral region of Lake Balaton, and kept in indoor aquaria filled with aerated, filtered Balaton water under 16–18 h light–dark cycle at room temperature (~16–20 °C) A week before testing, about 250 animals were separated into 15 L containers. In order to ensure/defilade their hideaway, which is characteristic of their lifestyle, small (diameter: 10 mm) ceramic tubes were placed/set into the containers. The shrimps were fed on carrot *(Daucus carota subsp. Sativus)* ad libitum. Prior to the experiment the animals were starved for the day before the experiment.

### Chemicals

The formulated product of a commercially available insecticide APACS 50 WG (*clothianidin*) was obtained from Arysta Life Science (Budapest, Hungary), and the chemicals for the Bradford assay (Bradford 1976) were purchased from SIGMA.

### Locomotor activity assay

This assay was carried out with three groups, one control and two treated groups, in each group ten animals were selected. The concentration of APACS 50WG was 3.9 ng l^−1^ dissolved in Balaton water. The exposition lasted 3 h. Individual animals were placed in 6-well plates, and measurements of swimming behaviour were performed by video tracking system, taking short (90 s) records using a camera. The videos were analysed with *Fiji ImageJ 1.52p* software. The programme recorded the coordinates and automatically reconstructs the trajectories of the test animals swimming within the ring of the whole of the plate.

### MXR assay

The animals were incubated in filtered water containing 2 μM rhodamine B for 60 min (loading). This was followed by a short (3 min) washing period in Balaton water, which allowed the removal of excess dye from the surface of tissues. T0 animals (6 pieces) were immediately frozen providing full uploaded concentration of rhodamine B. The remaining animals (6 pieces) were divided into two groups, individually placed in 100–100 mL low profile bakers. The animals in the control group were kept in filtered Balaton water. The acute (direct) effects of the insecticide were studied on the second group of the animals by incubation in the formulated products (APACS, respectively) dissolved in filtered Balaton water. Treated and untreated animals were frozen following the exposition period (3 h) until further processing. We applied the “accumulation” method of MXR assay (Vehovszky et al. 2018, Smital et al. 2003) where *rhodamine B* served as a model P-gp substrate. The samples were homogenized in a tissue layer (Tyssuelyzer LT, Qiagen) and centrifuged at 8000 g for 20 min at 4 °C (Biofuge Fresco, HERAUS). The fluorescence of the supernatant (250 μL) was finally measured at *λ*_ex_ = 535 nm and *λ*_em_ = 590 nm (Victor 3 plate reader, PerkinElmer) to determine the amount of accumulated *rhodamine B* in the tissue samples. The protein concentration of the tissue homogenates was also determined in parallel samples applying the Bradford assay (Bradford 1976). The *rhodamine B* accumulation data were finally expressed as fluorescence units per mg tissue protein.

### Statistical analysis

Data are presented as mean ± SE, and significance was calculated by using Student’s t-test.

## Results and discussion

**Fig. 1:**
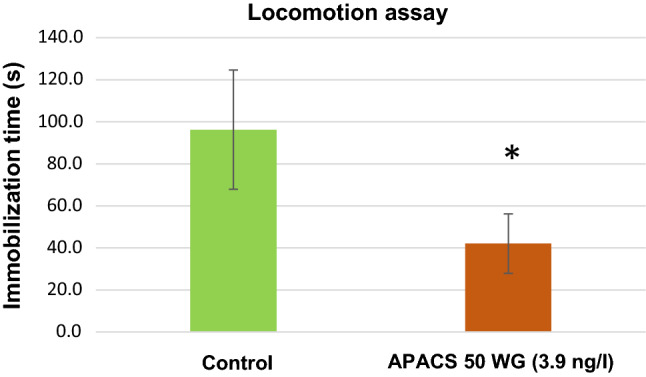
Effect of neonicotinoid insecticide (APACS 50WG, 3.9 ng/l) on locomotor activity of *D. villosus*. A. The swimming activity was highly impacted by insecticide exposure. The treated animals spent 58 ± 12% less immobility time during the experiment than the control individuals (error bars indicate standard error of mean), asterisk indicate (*p* < 0.01) significant differences between samples (Student’s *t*-test)

We conducted a locomotor activity test to detect toxic changes in swimming behaviour following APACS 50WG exposure. Short term application (3 h) of the insecticide (3.9 ng/l) elicited rapid movements described as a type A response (Benzidane et al. 2010). As a result, the treated animals spent less time in an immobile state (Fig. 1). In contrary, in the American cockroach, application of insecticide induced type B response, and cockroaches were immobilised (Tan et al. 2007). There are only a few reports about the behavioural effects of neonicotinoids on mammals. In an open field test, although the locomotor activities were not affected, the anxiety-like behaviours of the mice were elevated by clothianidin (Hirano et al. 2015).

**Fig. 2:**
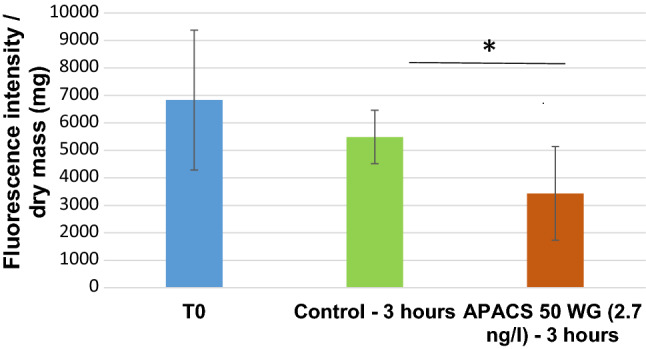
Effect of neonicotinoid insecticides on rhodamine accumulation in *D. villosus*. Fluorescence data of sample experiments with APACS 50WG (2.7 ng/l, 3 h treatment) show decreased rhodamine concentrations in tissues by 62 ± 16% compared with control group, indicating increased MXR activity. Summary of 6 parallel experiments, * (*p* < 0.05) indicate significant differences in rhodamine accumulation (Student’s *t*-test), error bars indicate standard error of mean.)

MXR activity was assessed on the differences of rhodamine accumulation data with and without APACS treatment—both were compared to T0 level. Based on our measurements (Fig. 2), it has been proven that APACS 50WG insecticide has an enhancement of MXR activity (chemostimulation). Though just a few literature data are available concerning the effects of neonicotinoids on the MXR system, in case of isolated mussel gill, it was found that the APACS 50WG acted as a substrate of MXR, while long-term treatment functioned as a chemostimulator (Vehovszky et al. 2018). We conclude that at a low concentration of neonicotinoid the oxidative damage evoked by the neonicotinoid impairs the intracellular compensatory mechanisms, yielding to increased activity against the neonicotinoid-induced toxicity. Regarding MXR activity, *Dikerogammarus* showed a one magnitude higher sensitivity (ng/l) to APACS 50WG than to mussels (Vehovszky et al. 2018).

In summary, APACS at very low doses alters the locomotor activity and it seems to be a potential substrate of the MXR mediated cellular efflux. Our results underline the importance of non-standard aquatic toxicity studies using *Gammarides*.
